# Sleep quality of patients with papillary thyroid carcinoma: a prospective longitudinal study with 5-year follow-up

**DOI:** 10.1038/s41598-022-23549-3

**Published:** 2022-11-05

**Authors:** Dae Lim Koo, Yangmi Park, Hyunwoo Nam, Young Jun Chai

**Affiliations:** 1 Department of Neurology, Seoul Metropolitan Government Seoul National University Boramae Medical Center and Seoul National University College of Medicine, Seoul, South Korea; 2 Department of Surgery, Seoul Metropolitan Government Seoul National University Boramae Medical Center, 39 Boramae-Gil, Dongjak-Gu, Seoul, 156-707 South Korea; 3 Transdisciplinary Department of Medicine & Advanced Technology, Seoul National University Hospital, Seoul, South Korea

**Keywords:** Head and neck cancer, Circadian rhythms and sleep

## Abstract

We evaluated the pre- and postoperative sleep quality of patients with newly diagnosed papillary thyroid carcinoma (PTC) who underwent thyroid surgery, and investigated the factors associated with persistent poor sleep quality. The Pittsburgh sleep quality index (PSQI), Epworth sleepiness scale, and Stanford sleepiness scale were used to estimate sleep quality and daytime sleepiness. Face-to-face surveys were conducted preoperatively, and 1, 4, and 10 months after thyroid surgery. The PSQI was administered during a telephone interview about after 5 years after surgery. Forty-six patients (mean age 47.3 ± 10.1 years) with PTC (11 males, 35 females) were included in this study. Twenty-one participants underwent lobectomy and 25 underwent total thyroidectomy. Preoperatively, 35 (76.1%) patients showed poor sleep quality. PSQI scores at postoperative 1, 4, and 10 months were significantly lower than preoperative scores (*p* < 0.001). Postoperative 5-year PSQI scores decreased significantly compared to the preoperative scores (*p* < 0.001). Patients newly diagnosed with PTC suffered from sleep disturbance before and after surgery for at least 10 months, recovering to a comparable rate of sleep disturbance with the general population by 5 years after surgery. Higher preoperative PSQI score was at risk for prolonged poor sleep quality in patients with PTC.

## Introduction

Thyroid cancer is the most common malignancy of the endocrine system. Due to improvements in diagnostic techniques, the worldwide incidence of thyroid cancer diagnosis has been increasing rapidly since the mid-1990s^1^. Treatment typically involves surgical intervention and radioactive iodine therapy to minimize the risk of recurrence and metastatic spread^2^.

The quality of life of patients with cancer links to their sleep quality^3^. Impaired sleep quantity or quality have been linked to numerous negative health outcomes, including insulin resistance, type 2 diabetes mellitus, cardiovascular disease, and mortality^4^. The prevalence of sleep disturbance in patients with cancer is 33–40%, which is about twice as high as the rate reported among the general population^5^. Physiological and psychological burdens related to a patient’s condition and treatment may increase their risk of poor sleep quality^6,7^. There are reports that sleep disturbance is associated with increased risk of thyroid cancer^8,9^. Likewise, good quality of sleep is reported to be a protective factor against thyroid cancer^10^.

While patients often complain of fatigue or sleep disturbance after thyroid surgery, there is a lack of research investigating sleep quality before and after surgery among patients with newly diagnosed thyroid cancer. In this study, we evaluated the preoperative and postoperative (PO) sleep quality of patients with newly diagnosed papillary thyroid cancer (PTC) and investigated the factors associated persistent poor sleep quality.

## Methods

### Study participants

We prospectively recruited subjects with newly diagnosed PTC at Seoul Metropolitan Government Seoul National University Boramae Medical Center between June 2016 and February 2017. Past medical history and clinicopathological features of thyroid cancer were obtained from the patient’s medical records. Written informed consent was obtained from all participants, and the Institutional Review Board at Seoul Metropolitan Government Seoul National University Boramae Medical Center approved this study (IRB No. 16-2016-61). The work described here was carried out in accordance with the Code of Ethics of the World Medical Association (Declaration of Helsinki) for experiments involving humans.

### Surgical procedures and postoperative follow-up

All participants underwent conventional open lobectomy (n = 21) or total thyroidectomy (n = 25). Thyroid surgeries were performed in a standardized method. Surgical extent and use of radioactive iodine treatment were determined in accordance with the 2015 American Thyroid Association Management Guidelines for Adult Patients with Thyroid Nodules and Differentiated Thyroid Cancer^11^. Thyroid hormone replacement therapy was initiated to maintain target thyroid stimulating hormone level below 0.1 mU/L for high-risk of recurrence patients and below 2.0 mU/L for low-risk patients^11^.

### Assessment of sleep

Following confirmation of thyroid carcinoma by fine needle aspiration or gun biopsy, three questionnaires about sleep quality were administered preoperatively, and at PO 1, 4, and 10-month timepoints. To assess the long-term sleep quality outcomes after thyroidectomy, the PSQI was administered via a telephone interview by an independent surveyor in April 2022, approximately 5 years after surgery.

The Pittsburgh Sleep Quality Index (PSQI) was used with permission from the authors to measure sleep quality and sleep patterns during the month before surgery (Supplementary Table S1)^12^. The PSQI contains self-rated questions addressing seven domains of sleep, which are evaluated on a 3-point scale (0–3 points). Total PSQI scores ranges from 0 to 21 with higher scores indicating worse sleep quality. The original authors proposed a cutoff value of 5 in the global score to distinguish poor sleepers (> 5) from good sleepers (≤ 5)^12^. In clinical practice, a cutoff score greater than 7 point has been reported to be more appropriate to determine poor sleep^13–16^. Therefore, we defined a ‘poor sleeper’ as a patient with a PSQI score greater than 7 points and a ‘persistent poor sleeper’ as a patient whose PSQI score at postoperative 10 months was greater than 7^17–19^. Daytime sleepiness was evaluated using the Epworth sleepiness scale (ESS; Supplementary Table S2) and the Stanford sleepiness scale (SSS; Supplementary Table S3)^20,21^. ESS scores range from 0 to 24, with a score greater than 10 indicating pathological sleepiness^22^. The SSS consists of a seven-point scale ranging from 1 (very alert) to 7 (very sleepy)^21^.

### Statistical analysis

The normality assumption of continuous variables was checked using the Kolmogorov–Smirnov test. The normality of the residuals was checked by linear regression analysis, to meet the assumptions of the model. An analysis of covariance (ANCOVA) model for continuous variables was used to compare sleep quality before surgery and at PO 1, 4, and 10 months. Cohen suggests that an effect size of 0.2–0.3 indicates a small effect, an effect size of around 0.5 indicates a medium effect, and an effect size of 0.8 or higher indicates a large effect. Binary outcomes were compared using χ^2^ tests or Fisher’s exact tests, depending on the distribution of variables according to the Kolmogorov–Smirnov test. Comparisons between groups were performed with the Mann–Whitney U test. Variables with a *p*-value < 0.1 on univariate regression were considered to be candidates for multivariate analysis. Odds ratios (ORs) and 95% CI were reported for significant differences. Statistical analyses were performed using SPSS statistical software version 21 (IBM Corp: IBM SPSS Statistics for Windows, Armonk, NY). Two-sided *p* values < 0.05 were considered statistically significant.

## Results

A total of 46 patients were included in the analysis. The mean age was 47.3 ± 10.1 and 11 male and 35 female patients were enrolled. No patient was on medication for mood disorder such as depression or anxiety disorder. The mean preoperative PSQI score was 9.5 ± 3.0, with 11 (23.9%) good sleepers, and 35 (76.1%) poor sleepers. The mean ESS and SSS scores were 6.7 ± 4.6 and 2.2 ± 0.8, respectively. Preoperatively, 46 patients completed all three questionnaires; at PO 1 month, only 29 patients completed all three questionnaires; at PO 4 and 10 months, 33 patients completed all three questionnaires; and 5.5 ± 0.2 years after preoperative evaluation, 33 patients completed long-term telephone PSQI questionnaires.

**Figure 1:**
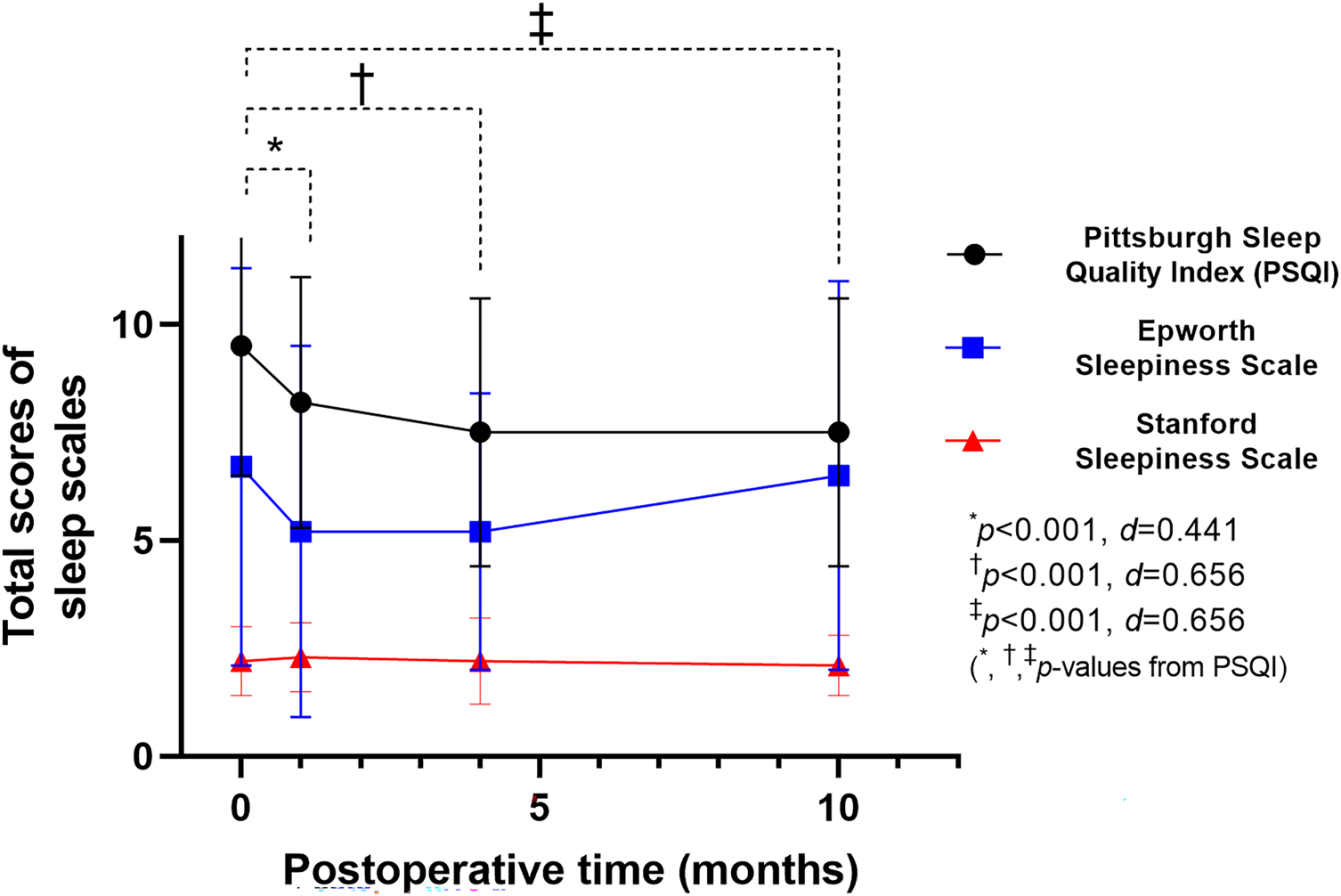
Repeated measures of sleep parameters before and after thyroidectomy with a 10-month follow-up. Error bars indicate ± standard deviations of the mean. *d* indicates Cohen’s *d.*

After adjusting for age, sex, BMI, and surgical extent, PO 1, 4, and 10-month PSQI scores decreased significantly compared with preoperative scores (8.2 ± 2.9, 7.5 ± 3.1, and 7.5 ± 3.1, respectively; Fig. 1). Compared to the preoperative PSQI scores, PO 5-year PSQI scores indicated a significant improvement in sleep quality (9.5 ± 3.0 to 5.4 ± 1.8, *p* < 0.001). Neither ESS or SSS scores changed significantly before and after surgery. Table 1 shows the clinicopathological characteristics of good and poor sleepers. There were no differences in any parameters between the two groups including age, gender, body mass index, preoperative thyroid stimulating hormone level, tumor size, nodal stage, postoperative risk, or the presence of radioactive iodine treatment.

**Table 1:** Clinicopathological characteristics of preoperative good and poor sleepers.

	Good sleeper^a^ (n = 11)	Poor sleeper^b^ (n = 35)	*p*-value^c^
Age, years	47.4 ± 10.6	47.3 ± 10.0	0.780
**Gender**			
Male	1 (9.1%)	10 (28.6%)	0.346
Female	10 (90.9%)	25 (71.4%)	
Body mass index, kg/m^2^	24.7 ± 4.7	25.4 ± 5.9	0.839
C**omorbidities**			
Hypertension	1 (9.1%)	8 (22.9%)	0.509
Diabetes mellitus	1 (9.1%)	5 (14.3%)	0.800
PSQI score	5.6 ± 1.1	10.7 ± 2.3	< 0.001
ESS score	4.9 ± 3.7	7.3 ± 4.7	0.105
SSS score	1.8 ± 0.6	2.4 ± 0.8	0.059
Preoperative TSH, uIU/mL	2.6 ± 1.8	2.3 ± 1.7	0.629
**Extent of surgery**			
Lobectomy	6 (54.5%)	15 (42.9%)	0.576
Total thyroidectomy	5 (45.5%)	20 (57.1%)	
**Tumor size, cm**	1.0 ± 0.5	1.3 ± 1.1	0.346
≤ 1.0 cm	7 (63.6%)	23 (65.7%)	0.919
> 1.0 cm	4 (36.4%)	12 (34.3%)	
**Nodal stage**			
N0	7 (63.6%)	18 (51.4%)	0.559
N1a	3 (27.3%)	13 (37.2%)	
N1b	1 (9.1%)	4 (11.4%)	
**ATA postoperative risk**			
Low	3	14	0.184
Intermediate	6	20	
High	2	1	
Radioactive iodine treatment	3 (27.3%)	10 (28.6%)	0.934

*PSQI* Pittsburgh sleep quality index, *ESS* Epworth sleepiness scale, *SSS* Stanford sleepiness scale, *TSH* thyroid stimulating hormone, *ATA* American thyroid association.

^a^Good sleeper was defined as a patient who had a PSQI score of 7 or less.

^b^Poor sleeper was defined as a patient who had a PSQI score greater than 7.

^c^*p*-values from the Mann–Whitney U test between good and poor sleepers.

Table 2 demonstrates the sleep quality scores of the lobectomy verses the total thyroidectomy groups. There were no differences between groups in the PSQI, ESS, or SSS scores at preoperative, and PO 1, 4, and 10 months. However, the mean PSQI score of the lobectomy group was lower than that of total thyroidectomy group (4.6 ± 1.4 vs 6.0 ± 1.8, p = 0.030) at the 5-year follow-up interview.

**Table 2:** Pre- and post-operative sleep disturbance scores in lobectomy and total thyroidectomy groups.

	Lobectomy (n = 21)	Total thyroidectomy (n = 25)	*p*-value^a^
**Preoperative**			
PSQI	8.6 ± 2.4	10.2 ± 3.4	0.120
ESS scores	5.7 ± 4.3	7.5 ± 4.7	0.090
SSS scores	2.3 ± 0.6	2.2 ± 1.0	0.131
**Postoperative 1 month**			
PSQI	8.0 ± 2.6	8.4 ± 3.1	0.880
ESS scores	4.6 ± 3.5	5.7 ± 4.8	0.682
SSS scores	2.3 ± 0.9	2.3 ± 0.8	0.901
**Postoperative 4 month**			
PSQI	7.2 ± 2.2	7.7 ± 3.6	0.726
ESS scores	4.1 ± 3.0	5.9 ± 3.2	0.143
SSS scores	2.3 ± 1.2	2.0 ± 0.7	0.161
**Postoperative 10 month**			
PSQI	7.4 ± 2.4	7.7 ± 3.6	0.986
ESS scores	6.5 ± 4.1	6.6 ± 4.9	0.825
SSS scores	2.3 ± 0.6	2.0 ± 0.8	0.266
**Postoperative 5 years**			
PSQI	4.6 ± 1.4	6.0 ± 1.8	0.030

*PSQI* Pittsburgh sleep quality index, *ESS* Epworth sleepiness scale, *SSS* Stanford sleepiness scale.

^a^*p*-values from the Mann–Whitney U test between lobectomy and total thyroidectomy groups.

**Figure 2:**
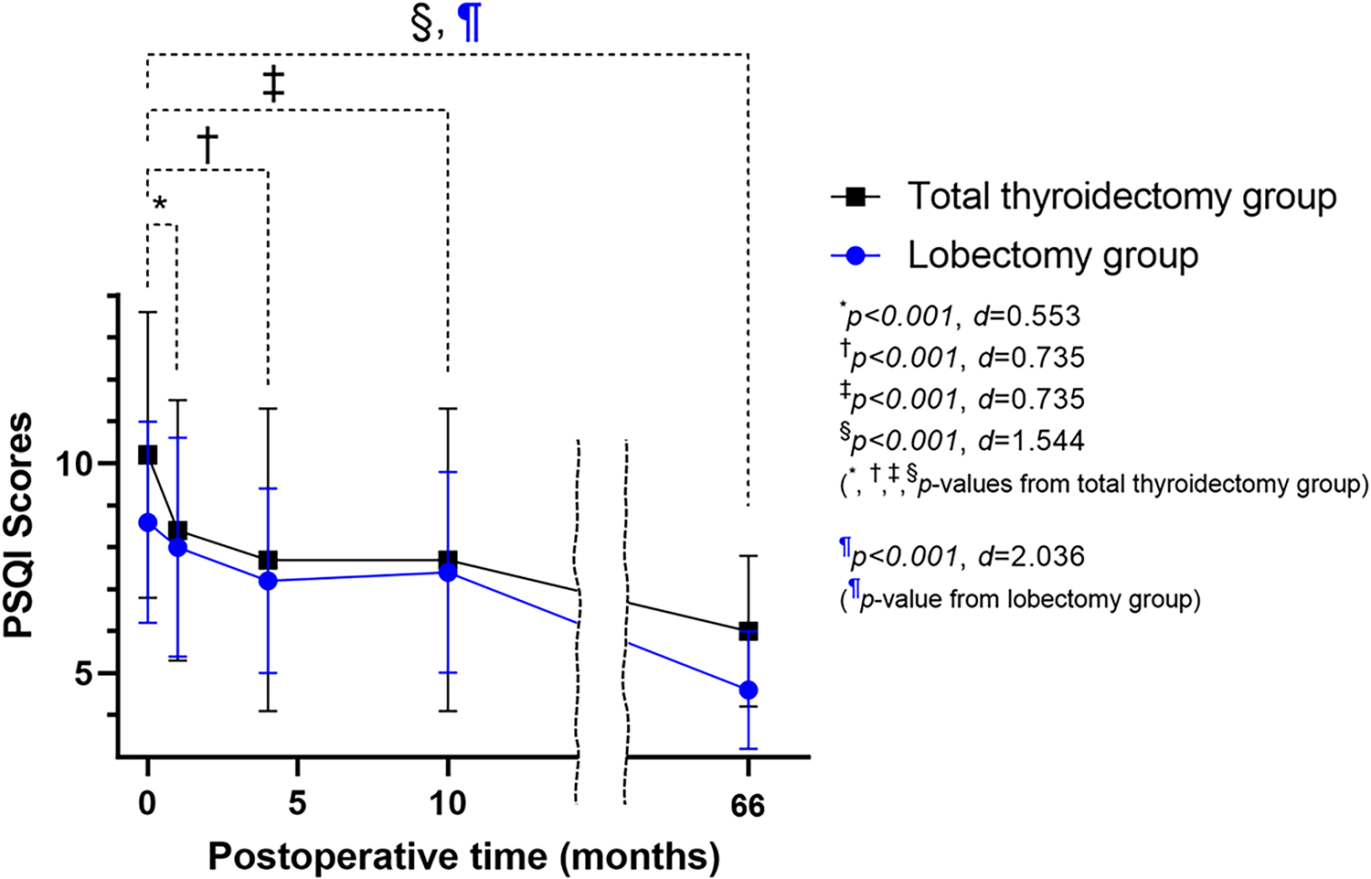
Comparison of PSQI scores in patients with thyroid cancer who underwent lobectomy vs total thyroidectomy. Error bars indicate ± standard deviations of the mean. *PSQI* Pittsburgh sleep quality index. *d* indicates Cohen’s *d.*

Figure 2 demonstrates the trend of the PSQI scores of the lobectomy and total thyroidectomy groups over time. In the lobectomy group, PSQI scores at preoperative and PO 1, 4, and 10 months were 8.6 ± 2.4, 8.0 ± 2.6, 7.2 ± 2.2, and 7.4 ± 2.4, respectively. No significant difference was observed between preoperative and 1, 4 and 10 months time points. Overall, the mean PO 5-year PSQI scores were significantly improved compared to the mean preoperative value (8.6 ± 2.4 vs 4.6 ± 1.4, *p* = 0.002). In the total thyroidectomy group, the preoperative PSQI scores before surgery and at PO 1, 4, and 10 months were 10.2 ± 3.4, 8.4 ± 3.1, 7.7 ± 3.6, and 7.7 ± 3.6, respectively. The PO 1, 4, and 10-month PSQI scores were lower than the preoperative PSQI scores (*p* < 0.001, *p* < 0.001, and *p* < 0.001). Mean PO 5-year PSQI scores were significantly improved compared to the preoperative scores (10.2 ± 3.4 to 6.0 ± 1.8, *p* < 0.001). The PSQI, ESS, and SSS scores of the six patients who underwent total thyroidectomy followed by radioactive iodine treatment are shown in Table 3.

**Table 3:** Pre- and post-operative sleep disturbance scores in the patients who underwent total thyroidectomy followed by ^131^I treatment.

Parameters	Preoperative	Postoperative 1 month	*p*-value^a^	Postoperative 4 month	*p*-value^b^	Postoperative 10 month	*p*-value^c^
PSQI scores	9.8 ± 3.6	8.9 ± 3.5	0.084	7.8 ± 4.1	0.073	8.9 ± 4.2	0.084
ESS scores	6.4 ± 2.2	5.1 ± 4.7	0.260	5.0 ± 2.5	0.063	6.5 ± 3.2	0.572
SSS scores	1.9 ± 0.5	2.1 ± 0.8	0.564	1.9 ± 0.5	1.000	2.1 ± 0.6	1.000

Values are express as mean ± SD.

*PSQI* Pittsburgh sleep quality index, *ESS* Epworth sleepiness scale, *SSS* Stanford sleepiness scale.

^a^*p*-values from the Mann–Whitney U test between preoperative and postoperative 1 month.

^b^*p*-values from the Mann–Whitney U test between preoperative and postoperative 4 month.

^c^*p*-values from the Mann–Whitney U test between preoperative and postoperative 10 month.

The clinicopathological features of non-persistent and persistent poor sleepers are compared in Table 4. Persistent poor sleepers showed higher PSQI scores at preoperative, and PO 1, 4, and 10 months compared to non-persistent poor sleepers (*p* = 0.022, *p* < 0.001, *p* = 0.002, and *p* < 0.001, respectively).

**Table 4:** Comparison of clinicopathological characteristics between non-persistent and persistent poor sleepers.

	Non-persistent poor sleeper^a^ (n = 14)	Persistent poor sleeper^b^ (n = 19)	*p*-value^c^
Age, years	50.2 ± 10.4	44.0 ± 8.7	0.100
**Gender**			
Male	3 (21.4%)	3 (15.8%)	0.464
Female	11 (78.6%)	16 (84.2%)	
Body mass index, kg/m^2^	24.1 ± 4.5	26.7 ± 7.5	0.464
**Comorbidities**			
Hypertension	1 (7.1%)	2 (10.5%)	0.464
Diabetes mellitus	1 (7.1%)	1 (5.3%)	0.957
Preoperative PSQI score	8.6 ± 3.2	10.7 ± 2.7	0.022
PSQI score at postoperative 1 month	5.9 ± 1.8	10.7 ± 2.1	< 0.001
PSQI score at postoperative 4 months	6.0 ± 2.6	9.8 ± 2.6	0.002
PSQI score at postoperative 10 months	5.0 ± 1.5	9.8 ± 2.0	< 0.001
ESS score at postoperative 10 months	5.1 ± 5.0	7.3 ± 3.5	0.682
SSS score at postoperative 10 months	1.9 ± 0.6	2.3 ± 0.7	0.166
Preoperative TSH, uIU/mL	2.7 ± 1.4	2.3 ± 1.1	0.421
**Extent of surgery**			
Lobectomy	5 (35.7%)	10 (52.6%)	0.630
Total thyroidectomy	9 (64.3%)	9 (47.4%)	
**Tumor size, cm**	1.0 ± 0.6	1.2 ± 0.9	0.274
≤ 1.0 cm	10 (71.4%)	11 (57.9%)	0.401
> 1.0 cm	4 (28.6%)	8 (42.1%)	
**Nodal stage**			
N0	7 (50.0%)	11 (57.9%)	0.901
N1a	5 (35.7%)	6 (31.6%)	
N1b	2 (14.3%)	2 (10.5%)	

*PSQI* Pittsburgh sleep quality index, *ESS* Epworth sleepiness scale, *SSS* Stanford sleepiness scale, *TSH* thyroid stimulating hormone.

^a^Non-persistent poor sleeper was defined as a patient who had a PSQI score of 7 or less at postoperative 10 months.

^b^Persistent poor sleeper was defined as a patient who had a PSQI score greater than 7 at postoperative 10 months.

^c^*p*-values from the Mann–Whitney U test between non-persistent and persistent poor sleepers.

Table 5 summarizes the results of univariate and multivariate logistic regressions of the factors associated with persistent poor sleep quality after surgery. Multivariate analysis revealed that older age (adjusted OR, 1.13; 95% CI 1.13–1.26; *p* = 0.032) and higher preoperative PSQI scores (adjusted OR, 1.46; 95% CI 1.06–2.01; *p* = 0.021) were risk factors for persistent poor sleep quality.

**Table 5:** Risk factors for persistent poor sleep quality after thyroid surgery.

	Univariate analysis			Multivariate analysis		
	OR	95% CI	*p-*value	OR	95% CI	*p-*value
Age	1.08	0.99–1.17	0.083	1.13	1.01–1.26	0.032
Women	2.91	0.45–8.74	0.261	–	–	–
BMI	1.08	0.95–1.23	0.259	–	–	–
Initial PSQI score	1.31	0.99–1.74	0.060	1.46	1.06–2.01	0.021
Initial ESS score	0.99	0.86–1.15	0.926	–	–	–
Initial SSS score	1.31	0.55–3.07	0.543	–	–	–
Hypertension	2.91	0.45–8.74	0.261	–	–	–
Diabetes mellitus	1.21	0.07–9.22	0.894	–	–	–
Preoperative TSH, mL/dL	2.62	0.21–12.08	0.452	–	–	–
Tumor size, cm	1.83	0.55–6.09	0.322			
Lateral lymph node metastasis	1.23	0.15–9.97	0.846	–	–	–
Total thyroidectomy	1.50	0.38–6.00	0.566			
Radioactive iodine treatment	1.06	0.23–4.94	0.943	–	–	–

Persistent poor sleeper was defined as a patient who had a PSQI score greater than 7 at postoperative 10 months.

*OR* odds ratio, *CI* confidence interval, *BMI* body mass index, *PSQI* Pittsburgh sleep quality index, *ESS* Epworth sleepiness scale, *SSS* Stanford sleepiness scale, *TSH* thyroid stimulating hormone.

## Discussion

This prospective longitudinal study investigated sleep quality before and after thyroid surgery among patients diagnosed with PTC, and explored the factors associated with persistent poor sleep quality in the target population. To the best of our knowledge, this is the first report to demonstrate the immediate and long-term impact of PTC and thyroid surgery on sleep quality among patients with PTC. Our results indicate that a considerable number of patients with PTC suffer from sleep disturbance before and after thyroid surgery, and that older age and higher preoperative PSQI scores are associated with persistent poor sleep quality.

Patients with thyroid cancer often complain of sleep disturbance after surgery. In fact, a cross-sectional study reported that poor sleep quality was more common among patients with thyroid cancer than in patients with benign thyroid disease, even after thyroid surgery^9^. However, because of its cross-sectional design, the previous study could not evaluate the impact of surgery on sleep quality. A large-scale longitudinal study which investigated the natural course of insomnia among patients with various types of cancer over an 18-month period reported that the incidence of insomnia peaked before surgery and dwindled after surgery over the subsequent 18 months^23^. Insomnia was more commonly observed among patients with many types of cancer including breast, prostate, gynecological, head and neck, and upper gastrointestinal tract cancers. The study showed that while the incidence of insomnia decreased over time after surgery, the incidence was still higher than that of the ‘non-cancer’ population. Likewise, in the current study, the mean PSQI score was highest before surgery and remained above 7 in the 10 months following surgery, although it tended to decrease after surgery.

In the current study, we found that sleep quality was significantly worse before thyroid surgery compared to after surgery. There are several factors which may be involved in the association between sleep disturbance and thyroid cancer. First, sleep disturbance itself may increase the risk of cancer. The prevalence of sleep disturbance is at least two times higher in patients with cancer than in the general population^5^. Our results revealed the prevalence of poor sleep quality was much higher among patients with PTC (PSQI > 5, 89.1%; PSQI > 7, 76.1%) than the general population (15.9–41.0%)^24,25^. Another explanation for the high prevalence of sleep disturbance among patients with thyroid cancer is that sleep deprivation may cause thyroid stimulating hormone elevation^26^ which may be associated with a greater likelihood of thyroid cancer^26–30^. However, in the current study, preoperative serum thyroid stimulating hormone (TSH) levels were not different between good and poor sleepers. Impaired immune function caused by sleep disturbance has been proposed as another possible risk factor for thyroid cancer^31^. Disrupted endocrine and physiologic circadian rhythms may lead to impaired circadian rhythm at the level of immune cells^32^. A recent study demonstrated that patients with well-differentiated thyroid cancer exhibit altered expression of clock genes in comparison with healthy controls or subjects with benign thyroid nodules^33^. Another study demonstrated alterations of clock genes, overexpression of *BMAL1,* and downregulation of *CRY2*, in patients with follicular thyroid carcinoma and PTC^34^.

Fear is another important potential cause of sleep disturbance in patients with thyroid cancer. There are various relevant types of fear such as fear of cancer diagnosis and fear of general anesthesia or surgery, as well as fear of postsurgical treatment including life-long medication, surgical complications, or recurrence. A recent study reported that anxiety in patients with thyroid cancer significantly improved after surgery^35^. PSQI scores decreased after surgery in that study. The authors believed that relief from fear of surgery or surgical complications was the key factor in the improvement in sleep quality after surgery. They suggested that because TSH levels are controlled after surgery with medication if needed, sleep disturbance may be caused by anxiety or fear, rather than the hormonal effects of the condition.

We hypothesized that when stratified by surgical type, sleep quality before and after surgery would be worse in the total thyroidectomy group compared to the lobectomy group. There are several reasons that patients undergoing total thyroidectomy may have more anxiety than those undergoing lobectomy prior to surgery. Total thyroidectomy is indicated for tumors larger than 4 cm, tumors with extensive lymph node involvement or distant metastasis, or for bilateral tumors, all of which are associated with unfavorable prognosis. In addition, there are complications which occur only after total thyroidectomy such as bilateral recurrent laryngeal nerve palsy, hypoparathyroidism, and life-long thyroid hormone replacement. During the preoperative period, concerns about poor prognosis and postoperative complications may aggravate the psychological fear of surgery among patients scheduled for total thyroidectomy. Contrary to our expectation, PSQI scores of total thyroidectomy group and lobectomy group were comparable during the pre- and postoperative period during the 10 months follow-up. This can be explained by the assumption that the number of the patients in the both groups were too small to show the difference and the concerns for thyroid surgery were alleviated after surgery.

The PSQI scores of the PTC patients 10 months after surgery remained higher than that of the normal population, which was reported to be 5.6 by a Korea Community Health Survey^25^. This suggests that anxiety among patients with PTC may continue after surgery. On the other hand, the PSQI scores decreased to 5.4, which is comparable to that of the general population, by the PO 5-year timepoint^25^. Furthermore, the PO 5-year PSQI scores indicate that the long-term sleep quality of the lobectomy group was significantly improved compared to the total thyroidectomy group. This suggests that sleep quality may improve as concern and anxiety is gradually alleviated over time, and the effect size may depend on the surgical extent.

Reports show that sleep disturbance may be associated with the initiation of radiotherapy or chemotherapy, and a high degree of sleep disturbance can be maintained over the treatment period with adjuvant therapies^9,36–38^. Radioactive iodine treatment is the most common β radiation nuclear medicine therapy. A study reported that mean PSQI scores increased from 7.6 to 8.8 after radioactive iodine treatment^9^. In the current study, among the six patients treated with total thyroidectomy followed by radioactive iodine therapy, the mean preoperative PSQI score was 9.8. At PO 1 month, the mean PSQI score reduced to 8.9, and further decreased to 7.8 at PO 4 months, before increasing again to 8.9 at PO 10 months. Our repeated measures of PSQI score data indicated that radioactive iodine treatment showed no harmful effect on sleep quality, although diet restriction and conditioning with severe hypothyroidism may have an unfavorable impact on sleep^39,40^.

Excessive daytime sleepiness, which is a cardinal feature of altered sleep status, is commonly reported in patients with cancer^41^. Hypersomnia has been associated with multiple factors including cancer types, advanced cancers, active chemotherapy, and side effects from antineoplastic therapy^5,42^. A previous study of patients with advanced cancers (head and neck, lung, breast, gynecological, genitourinary, gastrointestinal, etc.) demonstrated that 50% of patients showed cancer-related somnolence and mean ESS score was 11.2^43^. In this study, the reason why there was no significant change in the ESS and SSS scores was that preoperative scores (ESS 7.2, SSS 2.2) were already low. It shows patients were not in sleepy status before surgery, therefore, no improvement could be observed. Our data suggests that thyroid cancer might have a lower risk for daytime sleepiness, although hypersomnia in cancer patients has been associated with active chemotherapy, brain tumors, and advanced cancers^5,42,44^.

This study has several limitations. First, we did not apply scales to evaluate mood disorders such as anxiety or depression, which are important factors associated with sleep disturbance among cancer patients^9^. Although no patients were on medication for mood disorders during the study period, in-depth interview evaluations of mild mood disorders which do not require medication were not conducted. Second, we did not evaluate the sleep quality of study participants at regular intervals between PO 10 months and 5 years. Therefore, although we showed long-term sleep quality outcomes around 5 years after surgery, we were unable to ascertain the specific point at which the sleep quality of patients with PTC became comparable to that of the general population. Another limitation of this study was the relatively scarce number of patients and the lack of a control group. Comparing sleep quality of patients with PTC with that of patients with different cancers or patients without cancer may have further elucidated the impact of PTC on sleep disturbance in the target population. Further studies with a broader case series should be followed. Next, sleep characteristics, including sleep architecture and sleep apnea, were not assessed objectively using polysomnography in this study. As a recent study reported that moderate to severe obstructive sleep apnea may have a negative impact on cancer progression in patients with PTC^45^, further studies to elucidate the association between sleep apnea and the aggressiveness of thyroid cancer are required. Last, we could not determine whether PTC had a causative role in sleep disturbance or sleep disturbance had a causative role in PTC occurrence. In this study, preoperative questionnaires were administered when cytologic tests result confirmed PTC and surgery was planned. Therefore, the results reflect only the patient’s sleep quality during a period when they were experiencing fear while waiting for surgery. The use of a measurement tool that reflects sleep quality before recognition of the thyroid nodule or diagnosis of thyroid cancer may be required to determine the causative role of sleep disturbance on thyroid cancer, or vice-versa.

## Conclusions

Newly diagnosed patients with PTC suffered from sleep disturbance before and after surgery. High preoperative PSQI scores were associated with persistent poor sleep quality 10 months after surgery. The sleep quality of patients with PTC 5 years after surgery was comparable with that of the general population. Although the cause of sleep disturbance among patients with PTC remains unknown, physicians should be aware of the high incidence of sleep disturbance among this population and take measures to assess and manage sleep quality in patients with PTC.

## Supplementary Information

Supplementary Tables.

## Data Availability

The data that support the findings of this study are available from the corresponding author upon reasonable request.
