# Supplementary material for: Sleep quality of patients with papillary thyroid carcinoma: a prospective longitudinal study with 5-year follow-up
Source: Sci Rep. 2022 Nov 5;12:18823. doi: 10.1038/s41598-022-23549-3 (PMC9637156; doi:10.1038/s41598-022-23549-3)
Supplement: Supplementary file 1 — Supplementary Tables. [file 41598_2022_23549_MOESM1_ESM.docx]

**Sleep quality of patients with papillary thyroid carcinoma: A prospective longitudinal study with 5-year follow-up**

**Authors:**

Dae Lim Koo, MD,^1^ Yangmi Park, MD,^1^ Hyunwoo Nam, MD,^1^ Young Jun Chai, MD.^2^

**Affiliations:**

^1^Department of Neurology, Seoul Metropolitan Government Seoul National University Boramae Medical Center and Seoul National University College of Medicine, Seoul, South Korea

^2^Department of Surgery, Seoul Metropolitan Government Seoul National University Boramae Medical Center, Transdisciplinary Department of Medicine & Advanced Technology, Seoul National University Hospital, Seoul, South Korea

*** Corresponding author and person to whom reprint requests should be addressed**

Young Jun Chai, MD.

Department of Surgery, Seoul Metropolitan Government Seoul National University Boramae Medical Center, 39 Boramae-gil, Dongjak-gu, Seoul, 156-707, Republic of Korea

Tel: +82-2-870-3294

Fax: +82-2-831-0714

E-mail: [kevinjoon@naver.com](mailto:kevinjoon@naver.com)

**Supplementary Table 1. The Pittsburgh Sleep Quality Index**

Instructions: The following questions relate to your usual sleep habits during the past month only. Your answers should indicate the most accurate reply for the majority of days and nights in the past month. Please answer all questions.

During the past month,

| 1. When have you usually gone to bed? ___________________ | | | | |
| --- | --- | --- | --- | --- |
| 2. How long (in minutes) has it taken you to fall asleep each night? ___________________ | | | | |
| 3. When have you usually gotten up in the morning? ___________________ | | | | |
| 4. How many hours of actual sleep do you get at night? (This may be different than the number of hours you  spend in bed) ___________________ | | | | |
| 5. During the past month, how often have you had trouble sleeping because you… | Not during the past month (0) | Less than once a week (1) | Once or twice a week (2) | Three or more times week (3) |
| a. Cannot get to sleep within 30 minutes |  |  |  |  |
| b. Wake up in the middle of the night or early  morning |  |  |  |  |
| c. Have to get up to use the bathroom |  |  |  |  |
| d. Cannot breathe comfortably |  |  |  |  |
| e. Cough or snore loudly |  |  |  |  |
| f. Feel too cold |  |  |  |  |
| g. Feel too hot |  |  |  |  |
| h. Have bad dreams |  |  |  |  |
| i. Have pain |  |  |  |  |
| j. Other reason(s), please describe, including  how often you have had trouble sleeping  because of this reason(s): |  |  |  |  |
| 6. During the past month, how often have you taken medicine (prescribed or “over the counter”) to help you sleep? |  |  |  |  |
| 7. During the past month, how often have you had trouble staying awake while driving, eating meals, or engaging in social activity? |  |  |  |  |
| 8. During the past month, how much of a problem has it been for you to keep up enthusiasm to get things done? |  |  |  |  |
|  | Very good (0) | Fairly good (1) | Fairly bad (2) | Very bad (3) |
| 9. During the past month, how would you rate your sleep quality overall? |  |  |  |  |

**Supplementary Table 2. The Epworth sleepiness scale**

How likely are you to doze off or fall asleep in the following situations, in contrast to feeling just tired? This refers to your usual way of life in recent times. Even if you haven’t done some of these things recently try to work out how they would have affected you. Use the following scale to choose the most appropriate number for each situation:

| **Situation** | Chance of Dozing (0-3) |
| --- | --- |
| 1. Sitting and reading |  |
| 2. Watching TV |  |
| 3. Sitting, inactive in a public place (e.g. a theatre or a meeting) |  |
| 4. As a passenger in a car for an hour without a break |  |
| 5. Lying down to rest in the afternoon when circumstances permit |  |
| 6. Sitting and talking to someone |  |
| 7. Sitting quietly after a lunch without alcohol |  |
| 8. In a car, while stopped for a few minutes in the traffic |  |

0 = would **never** doze

1 = **slight chance** of dozing

2 = **moderate chance** of dozing

3 = **high chance** of dozing

***It is important that you answer each question as best you can.***

**Supplementary Table 3. The Stanford sleepiness scale**

This is a quick way to assess how alert you are feeling. If it is during the day when you go about your business, ideally you would want a rating of a one. Take into account that most people have two peak times of alertness daily, at about 9 a.m. and 9 p.m. Alertness wanes to its lowest point at around 3 p.m.; after that it begins to build again. Rate your alertness at different times during the day. If you go below a three when you should be feeling alert, this is an indication that you have a serious sleep debt and you need more sleep.

**An Introspective Measure of Sleepiness**

**The Stanford Sleepiness Scale**

| **Degree of Sleepiness** | Scale Rating |
| --- | --- |
| Feeling active, vital, alert, or wide awake | 1 |
| Functioning at high levels, but not at peak; able to concentrate | 2 |
| Awake, but relaxed; responsive but not fully alert | 3 |
| Somewhat foggy, let down | 4 |
| Foggy; losing interest in remaining awake; slowed down | 5 |
| Sleepy, woozy, fighting sleep; prefer to lie down | 6 |
| No longer fighting sleep, sleep onset soon; having dream-like thoughts | 7 |
| Asleep | X |
